# Clinical evaluation of deep learning-based risk profiling in breast cancer histopathology and comparison to an established multigene assay

**DOI:** 10.1007/s10549-024-07303-z

**Published:** 2024-04-09

**Authors:** Yinxi Wang, Wenwen Sun, Emelie Karlsson, Sandy Kang Lövgren, Balázs Ács, Mattias Rantalainen, Stephanie Robertson, Johan Hartman

**Affiliations:** 1 Department of Medical Epidemiology and Biostatistics, Karolinska Institutet, Stockholm, Sweden; 2 Stratipath AB, Nanna Svartz väg 4, Stockholm, 171 65 Sweden; 3 Department of Oncology-Pathology, Karolinska Institutet, Stockholm, Sweden; 4 Department of Clinical Pathology and Cancer Diagnostics, Karolinska University Hospital, Stockholm, Sweden; 5 MedTechLabs, BioClinicum, Karolinska University Hospital, Stockholm, Sweden

**Keywords:** Breast cancer, Deep learning, Digital pathology, Gene expression profiling, Prosigna

## Abstract

**Purpose:**

To evaluate the Stratipath Breast tool for image-based risk profiling and compare it with an established prognostic multigene assay for risk profiling in a real-world case series of estrogen receptor (ER)-positive and human epidermal growth factor receptor 2 (HER2)-negative early breast cancer patients categorized as intermediate risk based on classic clinicopathological variables and eligible for chemotherapy.

**Methods:**

In a case series comprising 234 invasive ER-positive/HER2-negative tumors, clinicopathological data including Prosigna results and corresponding HE-stained tissue slides were retrieved. The digitized HE slides were analysed by Stratipath Breast.

**Results:**

Our findings showed that the Stratipath Breast analysis identified 49.6% of the clinically intermediate tumors as low risk and 50.4% as high risk. The Prosigna assay classified 32.5%, 47.0% and 20.5% tumors as low, intermediate and high risk, respectively. Among Prosigna intermediate-risk tumors, 47.3% were stratified as Stratipath low risk and 52.7% as high risk. In addition, 89.7% of Stratipath low-risk cases were classified as Prosigna low/intermediate risk. The overall agreement between the two tests for low-risk and high-risk groups (*N* = 124) was 71.0%, with a Cohen’s kappa of 0.42. For both risk profiling tests, grade and Ki67 differed significantly between risk groups.

**Conclusion:**

The results from this clinical evaluation of image-based risk stratification shows a considerable agreement to an established gene expression assay in routine breast pathology.

**Supplementary Information:**

The online version contains supplementary material available at 10.1007/s10549-024-07303-z.

## Introduction

Based on classic clinicopathological variables, a significant proportion of estrogen receptor (ER)-positive and human epidermal growth factor receptor 2 (HER2)-negative early-stage breast cancer are categorized as clinically intermediate risk, thus providing limited information to guide adjuvant chemotherapy decisions. Prognostic risk profiling has become an integrated part of modern breast cancer diagnostics to provide additional risk information for this patient group for identifying patients where adjuvant chemotherapy can be omitted [1–3].

Among the established prognostic multiparameter diagnostic assays based on gene expression [4], the Prosigna assay (Prosigna Breast Cancer Prognostic Gene Signature Assay, Veracyte, South San Francisco, USA) is widely used and endorsed by national and international guidelines [5–8]. The Prosigna assay identifies intrinsic molecular subtypes (i.e., luminal A, luminal B, HER2-enriched and basal-like) and provides an individual risk of recurrence (ROR) score between 0 and 100 along with a three-tier risk category (low, intermediate, high) based on ROR score and nodal status. The Prosigna assay contributes with prognostic information for patients with early ER+/HER2- breast cancer and its efficacy has been demonstrated in several study populations [1, 9–14].

The diagnostic foundation with pathological assessment of the well-established prognostic variables such as tumor size, stage and tumor grade, is still an essential part of clinical decision making [15]. Among these, histologic grade is one of the most important prognostic factors for breast cancer [16, 17]. Approximately 50% of all [17–20], and around 60% of ER-positive/HER2-negative [1, 21] breast cancers are classified as histologic grade 2, which is a heterogenous group of tumors with variations in terms of aggressiveness and prognosis [22, 23], thus, associated with limited value to guide decisions on choice of therapy. A limitation in clinical decision-making is, despite the use of prognostic multigene assays, that tests such as Prosigna may classify up to 44% of histologic grade 2 tumors as intermediate risk [24], which does not add any clinically actionable information. In addition, the diagnostic multigene assays for breast cancer risk stratification show discordances in risk categorization between different tests [12, 25].

Digital pathology workflows are becoming standard practice and enable application of advanced image analysis in the clinical setting [26]. In addition, the recent evolution in deep learning, a field of artificial intelligence (AI), has further expanded the utility of machine learning techniques in computational pathology, making it possible to predict patient prognosis [27–32], response to neoadjuvant therapy [33], underlying molecular phenotypes [28, 34–37] or multigene assay results [38] in breast cancer using computer-based models to analyze and characterize histopathology whole slide images. Hence, computational pathology also plays a central role in precision medicine [26]. By leveraging grade-associated morphological features from hematoxylin and eosin (HE)-stained histopathology slides, deep learning-based image analysis has been shown to enable stratification of grade 2 tumors into two risk groups associated with risk of recurrence [27].

The novel AI-based precision diagnostic solution, Stratipath Breast (Stratipath AB, Solna, Sweden), is a commercial CE-IVD marked deep learning-based image analysis tool that utilizes digitized histopathological whole slide images to stratify intermediate risk patients in terms of risk of recurrence [29]. The test outputs a two-tier risk category. Compared with multigene assays, deep learning-based techniques have the strength of providing fast and cost-efficient solutions.

In this study, we provide the first clinical evaluation of the AI-based Stratipath Breast tool for image-based risk profiling where we compare it with an established multigene assay for risk stratification in a real-world breast cancer case series of clinically intermediate-risk ER+/HER2- tumors.

## Methods

### Patient inclusion and clinical data retrieval

This retrospective real-world case series consisted of 234 invasive breast tumors from patients with early ER-positive HER2-negative breast cancer, clinically assessed as intermediate-risk tumors and eligible for chemotherapy, diagnosed at Karolinska University Hospital and Södersjukhuset, Stockholm, Sweden. The case series represents consecutive tumors that had been analysed with the Prosigna assay in clinical routine at point of diagnosis between the years 2020 and 2022, to evaluate the patients’ risk of recurrence according to the Swedish national guidelines [39] and regional treatment guidelines. The guidelines recommend that gene expression-based analysis should be considered for postmenopausal patients with lymph node negative or 1–3 positive nodes (N0 or N1) and ER+/HER2- breast cancer where there is uncertainty about the tumor’s risk categorization prior to chemotherapy decisions. In addition, multigene assays were considered for luminal B-like tumors based on immunohistochemistry biomarker categorization to provide information for chemotherapy decision. The cohort has partly been expanded from Kjällquist et al. [24]. The Prosigna assay had been performed at the Department of Clinical Pathology and Cancer Diagnostics, Karolinska University Hospital, on sections from formalin-fixed paraffin-embedded (FFPE) breast cancer tissue blocks, according to the manufacturer’s instructions (Veracyte, South San Francisco, CA, USA) on the nCounter system (NanoString Technologies, Seattle, WA, USA) as part of clinical routine. Clinicopathological data including Prosigna results (intrinsic molecular subtype, ROR score (0-100) and risk group) were retrospectively retrieved from electronic records, along with the corresponding archived HE-stained, and parallel sectioned Ki67-stained, FFPE tissue slides.

Clinicopathological tumor data was retrieved from electronic records. Upon diagnosis, the routine biomarkers ER, progesterone receptor (PR), HER2 and Ki67, were assessed according to national guidelines [39]. Monoclonal rabbit anti-ER (clone SP1), anti-PR (clone 1E2), anti-HER-2/*neu* (clone 4B5) and anti-Ki67 (clone 30 − 9) antibodies were utilized according to the manufacturer’s instructions (BenchMark ULTRA Staining Module, Ventana Medical Systems, Arizona, USA). A positive ER or PR status was defined as > = 10% positive tumor nuclei. HER2 status was first determined by IHC, and tumors with 2 + score were subsequently evaluated for gene amplification by HER2 dual-probe in situ hybridization staining with VENTANA HER2 Dual ISH DNA Probe Cocktail assay (Roche Diagnostics, Rotkreutz, Switzerland) together with VENTANA Silver ISH DNP Detection kit and VENTANA Red ISH DIG detection kit according to the manufacturer’s instructions (BenchMark ULTRA IHC/ISH Staining Module, Ventana Medical Systems, Arizona, USA). Ki67 scores were reported as a continuous index that describe the percentage of positively stained tumor nuclei within a hotspot containing a minimum of 200 tumor cells, or from year 2022 as a global score across the entire tumor due to changes in the national guidelines.

Exclusion criteria are described in the consort diagram in Supplementary Fig. S1 and further in sections below. Only cases with complete results available from both the Prosigna and Stratipath Breast tests were included in the study comparisons.

### Ki67 global scoring

Due to the change in Ki67 scoring recommendations in 2022, all tumors were re-scored for Ki67 by the global scoring method using the open-source image analysis software QuPath [40]. All original Ki67 stained tissue slides were digitized in-house with a Hamamatsu NanoZoomer XR (Hamamatsu Photonics K.K., Shikuoka, Japan) at 40X magnification (0.226 μm/pixel). A protocol for digital Ki67 global scoring using QuPath was followed, described previously [41–43] and in accordance with recommendations from the International Ki67 in Breast Cancer Working Group (https://www.ki67inbreastcancerwg.org/; accessed on 18 July, 2023). The analysis was run on the entire invasive tumor area of the whole slide image (WSI) and output as a global Ki67 score (%). A few cases without digitized Ki67 slide available (*N* = 22) were manually evaluated by the global scoring method [44]. The cut-offs applied in the national guidelines were used for three-tier groups: Ki67-low (< 6%), Ki67-intermediate (6–29%), and Ki67-high (> 29%) [6].

### Stratipath Breast analysis

Stratipath Breast (Stratipath AB, Solna, Sweden) is a commercial CE-IVD marked deep learning-based image analysis tool for risk stratification of breast cancer patients. HE-stained slides of FFPE tissue sections were retrieved and subsequently digitized in-house with a Hamamatsu NanoZoomer XR at 40X magnification (0.226 μm/pixel). Each HE-stained WSI was analysed by Stratipath Breast (version 1.1). The image analysis model encompasses consecutive steps including quality assessment, cancer detection and risk stratification. Twenty-three images did not meet the intrinsic quality control of the Stratipath Breast analysis and were excluded from subsequent analysis (Supplementary Fig. S1). In addition, for two cases the WSIs were not available for Stratipath Breast analysis. All Stratipath Breast analysis reports underwent pathologist review to verify that adequate tumor area was analysed as part of quality control, and cases that did not meet this requirement were excluded (*N* = 14; Supplementary Fig. S1) and is in line with the manufacturer’s instructions for use (Stratipath AB). For each case Stratipath Breast provides a two-tier risk group; low risk or high risk, together with a continuous risk score (research use only).

### Statistical analysis

Descriptions of agreements between two risk stratification approaches were reported by the actual number and percentage, and Cohen’s kappa was used for two-group comparisons. The differences in distribution of patients belonging to each risk group, with respect to categorical clinical variables, were evaluated by the Fisher’s Exact test when the minimum number of patients in a subgroup was less than 5, or by the chi-square test otherwise. For comparing differences in continuous variables that were not assumed to be normally distributed, the Mann-Whitney U test (comparison across two groups) and Kruskal-Wallis test (comparison across more than two groups) were used. The correlation between continuous scores were calculated with Spearman correlation. All statistical analyses were 2-sided, and a *P* value of less than 0.05 was regarded as significant. The above statistical analyses were performed in IBM SPSS Statistics (version 28.0; IBM, Armonk, New York, USA). Changes in classification between tests were visualized by Sankey diagrams in https://jsfiddle.net.

## Results

### Patient characteristics

A total of 234 early-stage ER-positive/HER2-negative invasive breast tumors were included in the analyses of this study (Supplementary Fig. S1). The patients’ clinical characteristics and associated Prosigna results are summarized in Table 1. Most of the tumors were invasive carcinoma of no special type (NST) or mixed NST (79.5%) and 17.9% were invasive lobular carcinomas (ILC). The median Ki67 score was 24.5% (range 3.85–75.2%) by digital global scoring method. Out of all included tumors, the Prosigna assay classified 76 (32.5%), 110 (47.0%) and 48 (20.5%) tumors as low, intermediate and high risk, respectively. The median ROR score was 47 with a range from 3 to 84. The Prosigna intrinsic molecular subtypes were distributed as follows: 127 (54.3%) luminal A, 107 (45.7%) luminal B, 0 (0%) HER2-enriched and 0 (0%) basal-like. Notably, one patient had more than three lymph node metastases.

**Table 1:** Patient characteristics for all included cases and grade 2 cases

	All	Grade 2
	Frequency (%)	Frequency (%)
N	234	176
*Age, mean (Std Dev)*	65.10 (7.88)	65.11 (8.155)
*Primary tumor*		
No	5 (2.1%)	4 (2.3%)
Yes	229 (97.9%)	172 (97.7%)
*Bilateral breast cancer*		
No	225 (96.2%)	170 (96.6%)
Yes	9 (3.8%)	6 (3.4%)
*Histologic grade*		
Grade 1	12 (5.1%)	0 (0%)
Grade 2	176 (75.2%)	176 (100%)
Grade 3	46 (19.7%)	0 (0%)
*ER %, median (range)*	99 (20–100)	99 (20–100)
*ER status*		
Positive	234 (100%)	176 (100%)
*PR %, median (range)*	60 (0-100)	60 (0-100)
*PR status*		
Negative	56 (23.9%)	48 (27.3%)
Positive	178 (76.1%)	128 (72.7%)
*HER2 status*		
Negative	234 (100%)	176 (100%)
*Ki67%, median (range)* ^*a*^	24.49 (3.85–75.21)	21.19 (4.0-71.72)
*Ki67 status* ^*a*^		
Low	5 (2.1%)	3 (1.7%)
Intermediate	151 (64.5%)	126 (71.6%)
High	78 (33.3%)	47 (26.7%)
*Tumor size*		
<=20 mm	166 (70.9%)	122 (69.3%)
> 20 mm	67 (28.6%)	53 (30.1%)
N/A	1 (0.4%)	1 (0.6%)
*pT status*		
pT1	166 (70.9%)	122 (69.3%)
pT2	62 (26.5%)	48 (27.3%)
pT3	5 (2.1%)	5 (2.8%)
N/A	1 (0.4%)	1 (0.6%)
*Lymph node status*		
Negative	211 (90.2%)	158 (89.8%)
Positive	20 (8.5%)	16 (9.1%)
N/A	3 (1.3%)	2 (1.1%)
*pN status*		
pN0	211 (90.2%)	158 (89.8%)
pN1	19 (8.1%)	15 (8.5%)
pN2	1 (0.4%)	1 (0.6%)
N/A	3 (1.3%)	2 (1.1%)
*Histologic subtype*		
ILC	42 (17.9%)	39 (22.2%)
IMC	3 (1.3%)	2 (1.1%)
Mixed NST	7 (3.0%)	5 (2.8%)
NST	179 (76.5%)	129 (73.3%)
Other	3 (1.3%)	1 (0.6%)
*Prosigna ROR score, median (range)*	47 (3–84)	45 (3–80)
*Prosigna risk group*		
Low	76 (32.5%)	66 (37.5%)
Intermediate	110 (47.0%)	83 (47.2%)
High	48 (20.5%)	27(15.3%)
*Prosigna intrinsic subtype*		
Luminal A	127 (54.3%)	109 (61.9%)
Luminal B	107 (45.7%)	67 (38.1%)

ER = estrogen receptor, HER2 = human epidermal growth factor receptor 2, ILC = invasive lobular carcinoma, IMC = invasive mucinous carcinoma, N/A = not available, NST = invasive carcinoma of no special type, PR = progesterone receptor, pN = pathological N stage for regional lymph nodes according to TNM 8, pT = pathological T stage for invasive tumor according to TNM 8 (TNM classification of malignant tumors, 2017, 8th Ed), ROR = risk of recurrence

^a^Ki67 global scoring method

### Comparison between the tests for risk stratification

**Fig. 1:**
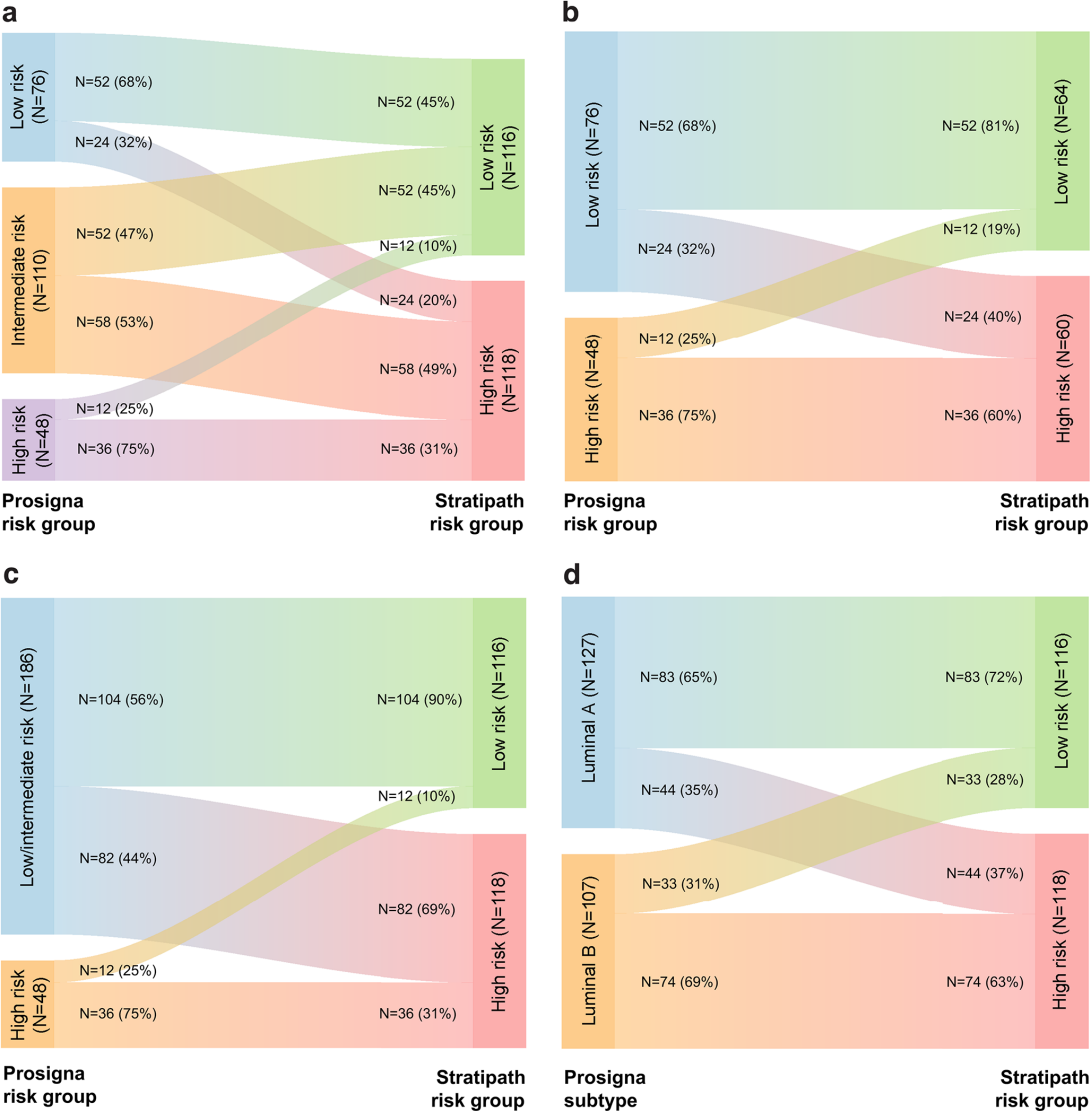
Sankey diagram of the re-classification of risk group between methods. Prosigna risk group vs. Stratipath risk group (**a**). Prosigna risk group (low and high) vs. Stratipath risk group (**b**). Prosigna risk group (low/intermediate and high) vs. Stratipath risk group (**c**). Prosigna intrinsic subtype vs. Stratipath risk group (**d**)

The Stratipath Breast analysis identified 116 (49.6%) tumors as low risk and 118 (50.4%) as high risk. Among Prosigna intermediate-risk tumors, 52 (47.3%) were stratified as low risk and 58 (52.7%) as high risk by Stratipath Breast (Fig. 1A; Table 2). In addition, 24 (31.6%) of the 76 Prosigna low-risk cases were upgraded as high-risk by Stratipath Breast, whereas 12 (25.0%) of the 48 Prosigna high-risk cases were downgraded by Stratipath Breast (Fig. 1B; Tables 2 and 3). The overall agreement between the two tests for low-risk and high-risk groups was 71.0%, with a Cohen’s kappa of 0.42. Prosigna intermediate-risk results were not included in the overall agreement estimate as it is non-informative for treatment decision making. However, when grouping Prosigna low and intermediate risk together, out of the 116 Stratipath low-risk cases 104 (89.7%) were Prosigna low/intermediate risk and 12 (10.3%) were high risk (Fig. 1C, Supplementary Table S1).

**Table 2:** Comparison of agreement in risk stratification between Stratipath Breast risk group and Prosigna risk group

		Prosigna risk group			
		Low	Intermediate	High	Total
Stratipath risk group	Low	52 (68.4%)	52 (47.3%)	12 (25%)	116 (49.6%)
	High	24 (31.6%)	58 (52.7%)	36 (75%)	118 (50.4%)
	Total	76 (100%)	110 (100%)	48 (100%)	234 (100%)

**Table 3:** Comparison of agreement in risk stratification between Stratipath Breast risk group and Prosigna risk group for low and high risk only

		Prosigna risk group		
		Low	High	Total
Stratipath risk group	Low	52 (68.4%)	12 (25%)	64 (51.6%)
	High	24 (31.6%)	36 (75%)	60 (48.4%)
	Total	76 (100%)	48 (100%)	124 (100%)

Among the 176 histologic grade 2 tumors, 97 (55.1%) and 79 (44.9%) were stratified as Stratipath low risk and high risk, respectively, whereas 66 (37.5%), 83 (47.2%) and 27 (15.3%) were stratified as Prosigna low, intermediate and high risk, respectively (Supplementary Table S2). The agreement between the two tests for low-risk and high-risk groups among grade 2 cases was 68.8%, with a Cohen’s kappa of 0.39. The grade 2 cases showed similar proportions of discordant risk categories as among all cases (Supplementary Table S3).

In total 36 cases showed two-level discordant risk category (low and high) by the two tests (Supplementary Table S4). There were 24 Stratipath-high, Prosigna-low cases, which all were grade 2 or 3, node negative and luminal A. Among the 12 Stratipath-low, Prosigna-high cases there was a mix of all grades, all but one case was luminal B, and there was a higher proportion of invasive lobular carcinoma (ILC; 33.3%) and invasive mucinous carcinoma (IMC; 16.7%) than in the opposite two-level discordant group (16.7% ILC and 0% IMC). Upon review, two of the Stratipath-low, Prosigna-high cases had incorrectly reported tumor size, which may have altered the ROR score and Prosigna risk category if re-tested.

### Clinicopathological characteristics across Stratipath risk groups

**Fig. 2:**
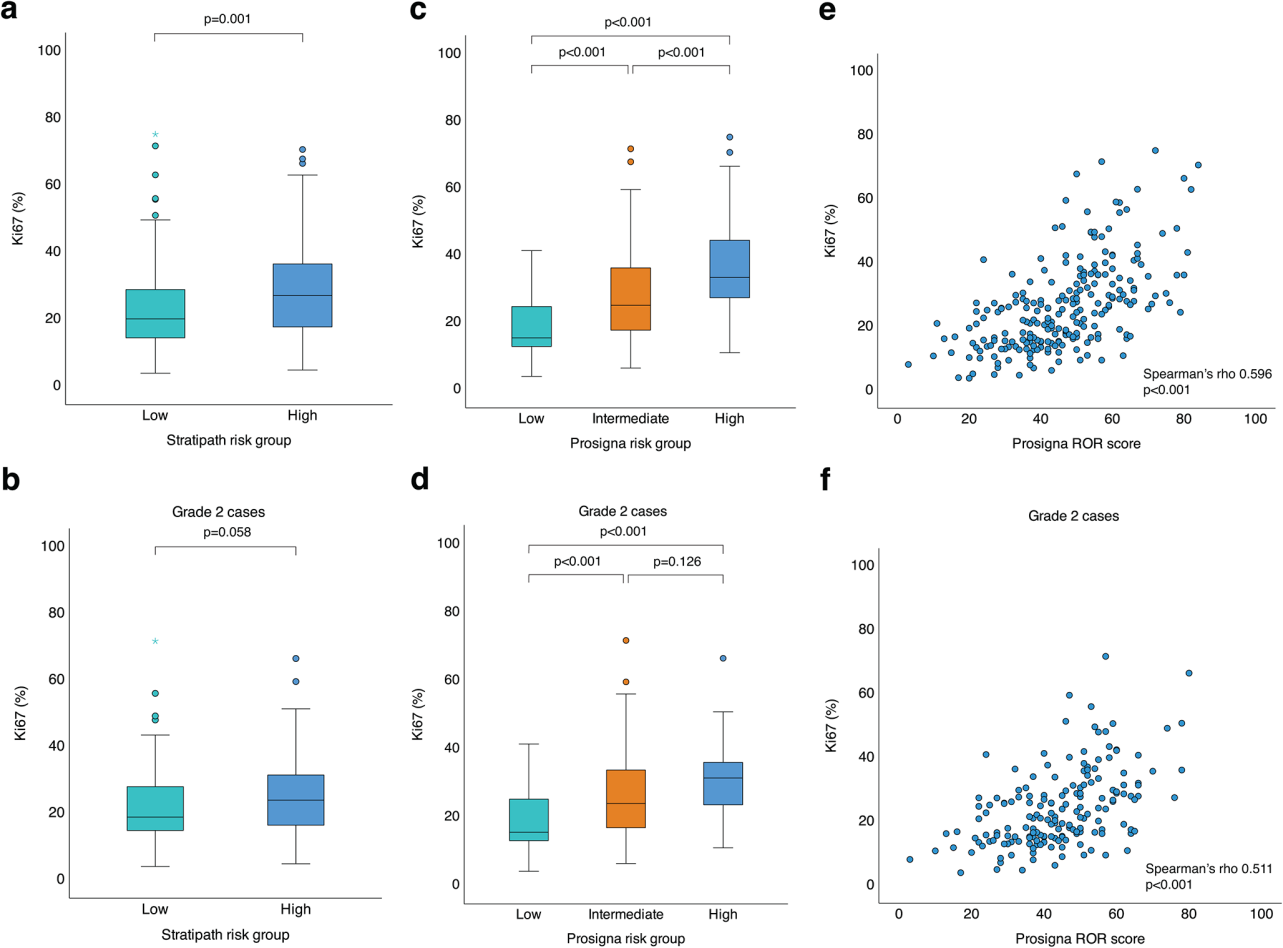
Comparison of Ki67 score across risk groups and correlation to Prosigna risk of recurrence (ROR) score. Significant difference in distribution of Ki67 score across Stratipath Breast risk groups (*p* = 0.001, Mann-Whitney U test). Box plot illustrating median, interquartile range and range (**a**). No difference in Ki67 score between Stratipath Breast risk groups among grade 2 cases (*p* = 0.058, Mann-Whitney U test) (**b**). Significant difference in distribution of Ki67 score across Prosigna risk groups (adjusted *p* < 0.001, between all three groups, Kruskal-Wallis test) (**c**). Distribution of Ki67 score across Prosigna risk groups for grade 2 cases showed a significant difference between low vs. intermediate risk (adjusted *p* < 0.001) and low vs. high risk (adjusted *p* < 0.001), but not between intermediate vs. high risk (adjusted *p* = 0.126, Kruskal-Wallis test) (**d**). Significant correlation between Ki67 score and Prosigna ROR score (Spearman’s rho 0.596, *p* < 0.001) (**e**). Significant correlation between Ki67 score and Prosigna ROR score for grade 2 cases (Spearman’s rho 0.511, *p* < 0.001) (**f**)

When comparing the distribution of clinical variables in each risk group, there was a significant difference in distribution of grade (*p* < 0.001), Ki67 status (*p* = 0.004), histologic subtype (*p* = 0.002) and intrinsic subtype (*p* < 0.001) across Stratipath risk groups, but no difference regarding PR status, lymph node status or tumor size (Table 4). The majority of grade 1 (10 of 12) and grade 3 (37 of 46) tumors were stratified as Stratipath low risk and high risk, respectively. There was a significant difference in the distribution of Ki67 score across Stratipath risk groups, with higher Ki67 scores in the high-risk than the low-risk group (Mann-Whitney U test *p* = 0.001; Fig. 2A). Among grade 2 cases, no difference in Ki67 score was observed between Stratipath risk groups (Mann-Whitney U test *p* = 0.058; Fig. 2B). For the group of grade 2 tumors, only histologic subtype (*p* = 0.010) and intrinsic subtype (*p* < 0.001) differed significantly between the Stratipath risk groups (Supplementary Table S5). ILC accounted for 17.9% of the tumors and 29 of the 42 (69.0%) ILC tumors were classified as low risk by Stratipath Breast, with even higher proportion among grade 2 cases (22.2% ILC and 74.4% of ILC as low risk). Among Prosigna intermediate-risk cases, a significant difference between Stratipath low-risk and high-risk groups was identified for grade (*p* = 0.002) and lymph node status (*p* = 0.013; Supplementary Table S6).

**Table 4:** Difference of distribution between risk groups for Stratipath Breast and Prosigna per clinicopathological characteristic

	Stratipath risk group				Prosigna risk group				
	**Low**	**High**	**Total**	p-value	**Low**	**Intermediate**	**High**	**Total**	p-value
*Histologic grade*				< 0.001^a^*					< 0.001^b^*
1	10	2	12		5	5	2	12	
2	97	79	176		66	83	27	176	
3	9	37	46		5	22	19	46	
Total	116	118	234		76	110	48	234	
*PR status*				0.321^a^					0.011^a^*
Negative	31	25	56		26	17	13	56	
Positive	85	93	178		50	93	35	178	
Total	116	118	234		76	110	48	234	
*Ki67 status* ^*c*^				0.004^b^*					< 0.001^b^*
Low	4	1	5		5	0	0	5	
Intermediate	84	67	151		65	70	16	151	
High	28	50	78		6	40	32	78	
Total	116	118	234		76	110	48	234	
*Tumor size*				0.918^a^					0.001^a^*
<=20 mm	83	83	166		45	90	31	166	
> 20 mm	33	34	67		31	19	17	67	
Total	116	117	233		76	109	48	233	
*Lymph node status*				0.154^a^					0.059^b^
Negative	102	109	211		73	98	40	211	
Positive	13	7	20		3	9	8	20	
Total	115	116	231		76	107	48	231	
*Histologic subtype*				0.002^b^*					0.102^b^
NST	82	97	179		53	89	37	179	
ILC	29	13	42		21	14	7	42	
Mixed NST	2	5	7		2	4	1	7	
IMC	3	0	3		0	1	2	3	
Other	0	3	3		0	2	1	3	
Total	116	118	234		76	110	48	234	
*Prosigna subtype*				< 0.001^a^*					< 0.001^b^*
Luminal A	83	44	127		76	49	2	127	
Luminal B	33	74	107		0	61	46	107	
Total	116	118	234		76	110	48	234	

ILC = invasive lobular carcinoma, IMC = invasive mucinous carcinoma, NST = invasive carcinoma of no special type, PR = progesterone receptor

^a^Chi-Square test; ^b^Fisher Exact test. All statistical tests are two-sided. *Significance at a *p* < 0.05 level. ^c^Ki67 global scoring method

Among the 116 Stratipath low-risk cases, 88 (75.9%) were Ki67-low/intermediate and 28 (24.1%) were Ki67-high (Supplementary Table S7). In total, 37.6% of all cases were both Stratipath low risk and Ki67-low/intermediate. Furthermore, 95.5% of the Stratipath low-risk group with Ki67-low/intermediate was Prosigna low or intermediate risk (Supplementary Table S7). Turning to the Stratipath high-risk group, two of the 50 (4.0%) Ki67-high cases were classified as Prosigna low risk (Supplementary Table S8).

### Clinicopathological characteristics across Prosigna risk groups

Across Prosigna risk groups, a significant difference in distribution of grade (*p* < 0.001), PR status (*p* = 0.011), Ki67 status (*p* < 0.001), tumor size (*p* = 0.001) and intrinsic subtype (*p* < 0.001) was observed (Table 4) and Ki67 status, tumor size, lymph node status and intrinsic subtype all remained significant among grade 2 cases (Supplementary Table S5). There was no difference in the distribution of histologic subtype between Prosigna risk groups.

Regarding Ki67 score, a significant difference in distribution of Ki67 score across all Prosigna risk groups was observed (Kruskal-Wallis test adjusted *p* < 0.001; Fig. 2C). Among grade 2 cases there was a difference in distribution of Ki67 score across the three Prosigna risk groups (Kruskal-Wallis test *p* < 0.001) with a significant difference between low vs. intermediate risk (adjusted *p* < 0.001) and low vs. high risk (adjusted *p* < 0.001), but not between intermediate vs. high risk (adjusted *p* = 0.126; Fig. 2D). In addition, Ki67 score showed a significant correlation with ROR score (Spearman’s rho 0.596, *p* < 0.001; Fig. 2E), also among grade 2 cases (Spearman’s rho 0.511, *p* < 0.001; Fig. 2F).

### ROR score and intrinsic subtype across Stratipath risk groups

**Fig. 3:**
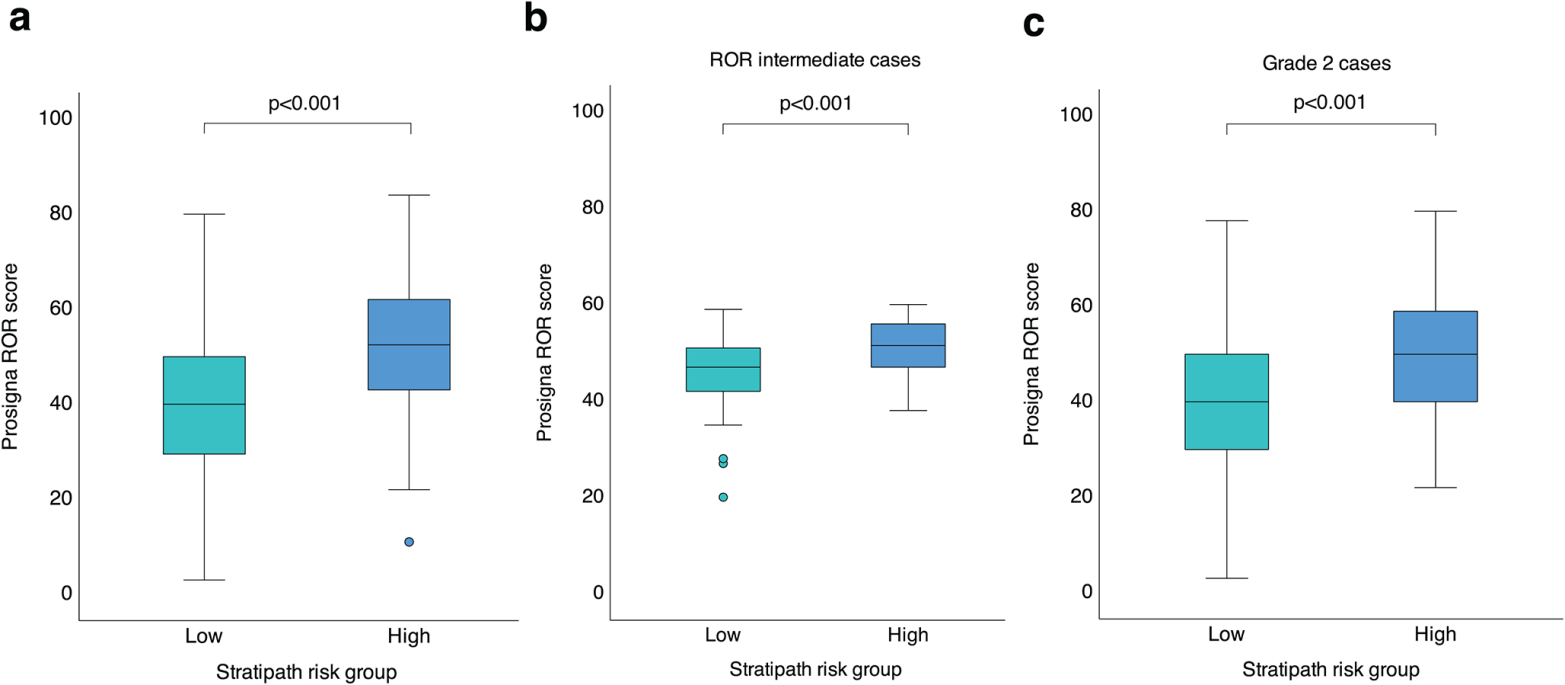
Difference in risk of recurrence (ROR) score across Stratipath risk groups. Higher ROR score in Stratipath high risk group than low risk group (*N* = 234; Mann Whitney U test *p* < 0.001) (**a**). Higher ROR score in Stratipath high risk group than low risk group among ROR intermediate cases (*N* = 110; Mann Whitney U test *p* < 0.001) (**b**). Higher ROR score in Stratipath high risk group than low risk group among grade 2 cases (*N* = 176; Mann-Whitney U test *p* < 0.001) (**c**). Box plots illustrating median, interquartile range and range

ROR scores were higher in the Stratipath high-risk group compared to the low-risk group (*p* < 0.001), across all cases as well as in the Prosigna intermediate-risk group and among grade 2 cases (Fig. 3). Regarding the distribution of intrinsic subtypes, a total of 83 out of 127 (65.4%) luminal A cases were classified as Stratipath low risk and 74 of 107 (69.2%) luminal B cases as Stratipath high risk (Fig. 1D, Supplementary Table S9) and similar results were observed among grade 2 cases (Supplementary Table S10). A significant difference in distribution of Prosigna intrinsic subtypes across Stratipath risk groups and Prosigna risk groups was identified for all cases as well as for grade 2 cases (Chi-square test and Fisher exact test *p* < 0.001; Table 4, Supplementary Table S5).

## Discussion

In this study we show the first clinical comparison between the AI-based tool Stratipath Breast and the well-established multigene assay Prosigna for risk profiling of clinically intermediate-risk breast cancer. The agreement between the two tests reached 71.0% (Cohen’s kappa of 0.42) for classifying patients as low risk and high risk. This considerable overall agreement between Stratipath Breast and Prosigna, two methodologically different tests, is on a similar level to what has been observed previously with respect to agreement between different multigene assays [25]. In a comparison of multigene tests in the OPTIMA Prelim trial, the overall agreement between Prosigna risk group and Oncotype DX recurrence score (Exact Sciences, Madison, USA) was 81.0% for low and high risk groups and 77.5% for low/intermediate and high risk groups [25]. A disagreement in this range is expected since these assays are based on different models, gene sets and clinical variables. Although demonstrating robust prognostic value on a population level, discrepancies on the individual patient level are evident when different multigene assays are performed on the same tumor sample [1, 12].

Risk profiling of breast cancer, by e.g., the Prosigna assay, is currently an integrated part of clinical routine diagnostics for clinically intermediate-risk early breast cancer patients. This is crucial to avoid inadequate treatment, especially for ambiguous cases where traditional biomarkers are insufficient to predict if patients would benefit from e.g., additional adjuvant chemotherapy or could be spared chemotherapy. We observed a higher proportion of all cases classified as low risk by Stratipath Breast (49.6%) than by Prosigna (32.5%), which may impact treatment decision to spare patients of chemotherapy. However, as Prosigna also provides an intermediate-risk group, which in this study constituted of almost half of the cases (47%), the risk information remains inconclusive for these patients in guiding treatment decisions. Our findings also showed that Stratipath Breast classified a high proportion (47.3%) of the Prosigna intermediate-risk group as low risk. In addition, the majority (89.6%) of the Stratipath low-risk cases were found in the Prosigna low/intermediate-risk group, which is the patient group where adjuvant endocrine therapy alone is generally considered, depending on local routines. The Prosigna high-risk group is generally considered candidates for adjuvant chemotherapy. Furthermore, when assessing Ki67 as a clinical factor together with Stratipath Breast, 95.5% of the Stratipath low-risk with Ki67-low/intermediate cases were also Prosigna low/intermediate risk. This supports the use of additional AI-based risk profiling to identify those patients that can be spared from chemotherapy, assuming that other risk factors are in alignment. Furthermore, differences in intrinsic subtype were observed not only in the Prosigna risk groups but also between Stratipath risk groups with higher proportion of luminal A tumors in the low-risk and luminal B tumors in the high-risk group.

Special resources at the individual pathology laboratory are required for running the Prosigna assay on the nCounter platform, including tissue preparation by macrodissection of invasive tumor region and sectioning prior to analyses. In comparison, Stratipath Breast is a fully automated decision support tool which operates on digitized routine HE-stained slides, thus, limited additional workload apart for the digitization of routine slides is required, ensuring a considerably reduced turnaround time and cost.

The heterogeneous nature of breast cancer and especially inter-tumoral heterogeneity of histologic grade 2 tumors has been illustrated by gene expression analysis (DNA microarray), which shows that these tumors constitute of a mixture of gene expression patterns found in grade 1 and 3 tumors [22]. The gene expression signature, Genomic Grade Index, was further developed and has shown prognostic potential to more accurately divide histologic grade 2 into a low- and high-risk group associated with risk of recurrence [22, 23, 45]. Leveraging the capacity of automated feature extraction by deep learning, studies have shown that it is feasible to predict RNA expression profiles [34, 46], DNA mutations [47], intra-tumoral heterogeneity [31] or intrinsic subtypes [36] directly from HE-stained slides. Further, by leveraging grade-related feature extraction, deep learning has shown the ability to stratify grade 2 tumors into a low- and high-risk group [27].

The AI-based image-analysis tool used in this study extracts information based on the morphological appearance in the HE-stained tumor WSI to determine the patient’s risk category. Histopathological variables including histologic subtype and tumor grade showed significant different distribution between low-risk and high-risk groups by Stratipath Breast. The deep learning model has capacity to capture a range of representations/features, that are grade-related, but other than the actual subcomponents routinely identified by the pathologist when determining Nottingham histologic grade (i.e., tubular formation, pleomorphism and mitotic count) [27]. Here, we showed that histologic grade was associated with both Stratipath Breast risk groups and Prosigna risk groups. The association with tumor grade has been shown for several multigene assays [48–51] and tumor grade has also been incorporated in prognostic index (Nottingham Px) for prognostic stratification of the clinically intermediate-risk group of breast cancer (node negative ER-positive/HER2-negative) [52].

To establish the risk category, Stratipath Breast utilizes only the WSI as input, i.e., without incorporating other clinical variables. On the contrary, several of the clinical variables shown to be significantly different between Prosigna risk groups in this study, are included in the ROR score. The PAM50 gene expression of the tumor sample designates the intrinsic subtype and is combined with a proliferation score and tumor size to calculate the ROR score [53]. Apart from the apparent methodological difference in the tests, it may be speculated that these differences in modalities could explain the discordances to some extent.

Differences in the prognostic performance between different assays can be explained by several factors, including different molecular markers included in the gene signature assays, where the Prosigna ROR score is largely determined by proliferative features whereas others are driven by ER-related features [51]. We found a significant correlation between the proliferation marker Ki67 and ROR score (Spearman’s rho 0.596) in this clinical case series, which is in line with previous findings [24]. A significant association between Prosigna risk categories and Ki67 status was observed in all patients, and in low- vs. intermediate-risk groups and in low- vs. high-risk groups in the grade 2 subgroup. For Stratipath Breast, Ki67 was significantly different between low- and high-risk groups when evaluating all patients but not in the subset of grade 2 tumors. This is not unexpected since the PAM50 gene assay incorporates eleven proliferation relative genes, and while Stratipath Breast does not explicitly include any information on proliferation, the AI-based approach has the capacity to capture proliferation associated morphological patterns in the WSIs.

Strengths of this study are that the CE-IVD marked commercial form of both tests was used and in a clinical case series from two sites in the intended patient population. However, the study has several limitations. One limitation to the study is the lack of follow-up information for prognostic comparisons but this was outside of the scope for this study and may instead be evaluated in future studies. Neither was treatment information available for evaluations of the effect on treatment decisions. Another limitation is that the Prosigna assay categorizes a relatively large proportion of the cases as intermediate risk, which is non-informative for decision making and was thus excluded in several comparisons focusing on the two-level agreement (low and high risk), and we note that this is an intrinsic limitation of the Prosigna assay.

To conclude, in this study of clinically assessed intermediate-risk ER-positive/HER2-negative breast cancers, we observed a considerable agreement between Prosigna and Stratipath Breast for low-risk and high-risk groups. This is the first study where a commercial multigene assay is compared to the image-based risk profiling tool Stratipath Breast. There was however a discrepancy of almost 30% between these two risk groups in the two risk profiling tests. Further studies with outcome data and impact on treatment decision are of value for clinical comparisons.

### Electronic supplementary material

Below is the link to the electronic supplementary material.

Supplementary Material 1

## Data Availability

The datasets analysed during the current study are not publicly available due to local privacy laws but are available from the corresponding author upon reasonable request.
