# Supplementary material for: Clinical evaluation of deep learning-based risk profiling in breast cancer histopathology and comparison to an established multigene assay
Source: Breast Cancer Res Treat. 2024 Apr 9;206(1):163–75. doi: 10.1007/s10549-024-07303-z (PMC11182789; doi:10.1007/s10549-024-07303-z)
Supplement: Supplementary file 1 — Supplementary Material 1 [file 10549_2024_7303_MOESM1_ESM.pdf]

Supplementary Information

for

Clinical evaluation of deep-learning-based risk profiling in breast cancer histopathology and comparison to an established multigene assay

Y. Wang, W. Sun, E. Karlsson, S. Kang Lövgren, B. Acs, M. Rantalainen, S. Robertson*, J. Hartman*

*Equal contribution

in

Breast Cancer Research and Treatment

Corresponding author: Stephanie Robertson, MD PhD.
Department of Oncology-Pathology, Karolinska Institutet, and Stratipath AB, Stockholm, Sweden.
Email: stephanie.robertson@ki.se

Supplementary Figures

Fig. S1 CONSORT diagram of study cohort.

Supplementary Tables

Table S1 Comparison of agreement in risk stratification between Stratipath Breast risk group and Prosigna risk group (low/intermediate vs high risk).
Table S2 Comparison of agreement in risk stratification between Stratipath Breast risk group and Prosigna risk group for grade 2 cases.
Table S3 Comparison of agreement in risk stratification between Stratipath Breast risk group and Prosigna risk group for low and high risk only among grade 2 cases.
Table S4 Cases with discordant risk category (low and high) between the two risk profiling tests.
Table S5 Difference of distribution between risk groups for Stratipath Breast and Prosigna per clinicopathological characteristic for grade 2 cases.
Table S6 Difference of distribution among Prosigna intermediate-risk cases between Stratipath Breast risk groups per clinicopathological characteristic.
Table S7 Crosstabulation of Ki67 status and Prosigna risk group for Stratipath Breast low-risk cases (N=116).
Table S8 Crosstabulation of Ki67 status and Prosigna risk group for Stratipath Breast high-risk cases (N=118).
Table S9 Comparison of agreement in risk stratification between Stratipath Breast risk group and Prosigna intrinsic subtype.
Table S10 Comparison of agreement in risk stratification between Stratipath Breast risk group and Prosigna intrinsic subtype for grade 2 cases.

Supplementary Figures

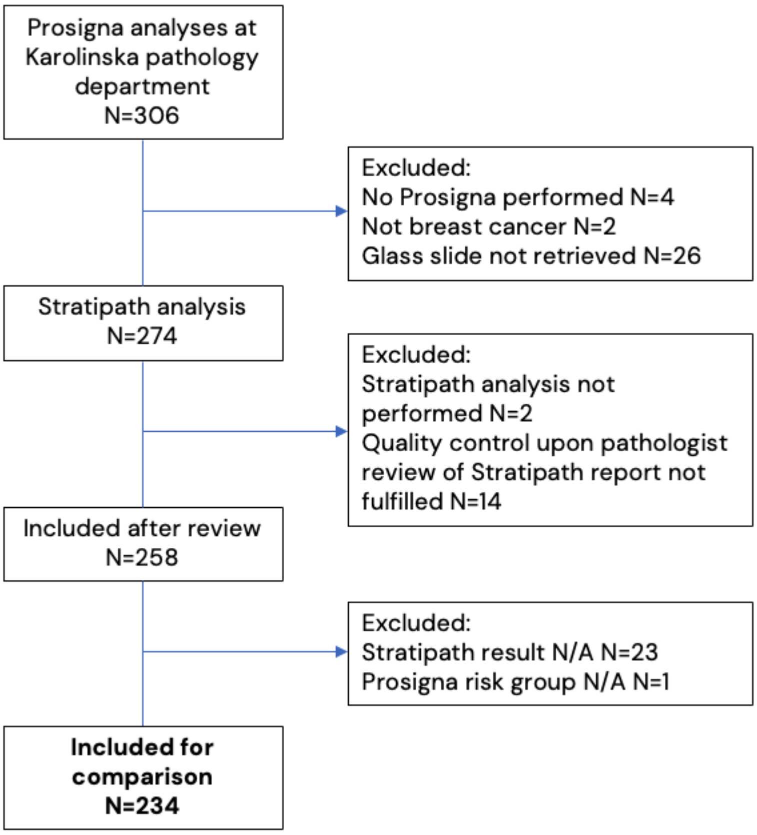

Fig. S1 CONSORT diagram of study cohort.

Supplementary Tables

Table S1 Comparison of agreement in risk stratification between Stratipath Breast risk group and Prosigna risk group (low/intermediate vs high risk).

		Prosigna risk group		
		**Low/Intermediate**	**High**	**Total**
**Stratipath risk group**	**Low**	104 (55.9%)	12 (25.0%)	116 (49.6%)
	**High**	82 (44.1%)	36 (75.0%)	118 (50.4%)
	**Total**	186 (100%)	48 (100%)	234 (100%)

Table S2 Comparison of agreement in risk stratification between Stratipath Breast risk group and Prosigna risk group for grade 2 cases.

		Prosigna Risk group			
		**Low**	**Intermediate**	**High**	**Total**
**Stratipath risk group**	**Low**	45 (68.2%)	44 (53.0%)	8 (29.6%)	97 (55.1%)
	**High**	21 (31.8%)	39 (47.0%)	19 (70.4%)	79 (44.9%)
	**Total**	66 (100%)	83 (100%)	27 (100%)	176 (100%)

Table S3 Comparison of agreement in risk stratification between Stratipath Breast risk group and Prosigna risk group for low and high risk only among grade 2 cases**.**

		Prosigna risk group		
		**Low**	**High**	**Total**
**Stratipath risk group**	**Low**	45 (68.2%)	8 (29.6%)	53 (57.0%)
	**High**	21 (31.8%)	19 (70.4%)	40 (43.0%)
	**Total**	66 (100%)	27 (100%)	93 (100%)

Table S4 Cases with discordant risk category (low and high) between the two risk profiling tests.

	Stratipath low | Prosigna high	Stratipath high | Prosigna low
N	12	24
Histologic grade		
1	1 (8.3%)	0 (0%)
2	8 (66.7%)	21 (87.5%)
3	3 (25.0%)	3 (12.5%)
Histologic subtype		
NST	6 (50.0%)	19 (79.2%)
ILC	4 (33.3%)	4 (16.7%)
Mixed NST	0 (0%)	1 (4.2%)
IMC	2 (16.7%)	0 (0%)
Other	0 (0%)	0 (0%)
Lymph node status		
Negative	10 (83.3%)	24 (100%)
Positive	2 (16.7%)	0 (0%)
Ki67 status[a]		
Low	0 (0%)	1 (4.2%)
Intermediate	4 (33.3%)	21 (87.5%)
High	8 (66.7%)	2 (8.3%)
Ki67 % median (range)	36.2 (17.0-75.2)	15.6 (4.8-41.4)
Prosigna subtype		
Luminal A	1 (8.3%)	24 (100%)
Luminal B	11 (91.7%)	0 (0%)
ROR score median (range)	66.50 (52-80)	36.50 (11-40)

[a]Ki67 global scoring method.
ILC=invasive lobular carcinoma, IMC=invasive mucinous carcinoma, NST=invasive carcinoma of no special type.

Table S5 Difference of distribution between risk groups for Stratipath Breast and Prosigna per clinicopathological characteristic for grade 2 cases.

	**Stratipath risk group**				**Prosigna risk group**				
	**Low**	**High**	**Total**	p-value	**Low**	**Inter-mediate**	**High**	**Total**	p-value
PR status				0.599[a]					0.074[a]
Negative	28	20	48		22	16	10	48	
Positive	69	59	128		44	67	17	128	
Total	97	79	176		66	83	27	176	
Ki67 status[c]				0.230[b]					<0.001[b]*
Low	2	1	3		3	0	0	3	
Intermediate	74	52	126		57	56	13	126	
High	21	26	47		6	27	14	47	
Total	97	79	176		66	83	27	176	
Tumor size				0.264[a]					0.035[a]*
<=20mm	71	51	122		40	65	17	122	
>20mm	26	27	53		26	17	10	53	
Total	97	78	175		66	82	27	175	
Lymph node status				0.928[a]					<0.001[b]*
Negative	87	71	158		64	75	19	158	
Positive	9	7	16		2	6	8	16	
Total	96	78	174		66	81	27	174	
Histologic subtype				0.010[b]*					0.271[b]
NST	64	65	129		45	65	19	129	
ILC	29	10	39		20	13	6	39	
Mixed NST	2	3	5		1	3	1	5	
IMC	2	0	2		0	1	1	2	
Other	0	1	1		0	1	0	1	
Total	97	79	176		66	83	27	176	
Prosigna subtype				<0.001[a]*					<0.001[b]*
Luminal A	71	38	109		66	41	2	109	
Luminal B	26	41	67		0	42	25	67	
Total	97	79	176		66	83	27	176	

[a]Chi-Square test; [b]Fisher Exact test. All statistical tests are two-sided. *Significance at a p <0.05 level. [c]Ki67 global scoring method.
ILC=invasive lobular carcinoma, IMC=invasive mucinous carcinoma, NST=invasive carcinoma of no special type, PR=progesterone receptor.

Table S6 Difference of distribution among Prosigna intermediate-risk cases between Stratipath Breast risk groups per clinicopathological characteristic.

	**Stratipath risk group**			
	**Low**	**High**	**Total**	p-value
Histologic grade				0.002[b]*
1	4	1	5	
2	44	39	83	
3	4	18	22	
Total	52	58	110	
PR status				0.985[a]
Neg	8	9	17	
Pos	44	49	93	
Total	52	58	110	
Ki67 status[c]				0.248[a]
Intermediate	36	34	70	
High	16	24	40	
Total	52	58	110	
Tumor size				0.297[a]
<=20mm	45	45	90	
>20mm	7	12	19	
Total	52	57	109	
Lymph node status				0.013[b]*
Neg	43	55	98	
Pos	8	1	9	
Total	51	56	107	
Histologic subtype				0.432[b]
NST	42	47	89	
ILC	8	6	14	
Mixed NST	1	3	4	
IMC	1	0	1	
Other	0	2	2	
Total	52	58	110	

[a]Chi-Square test; [b]Fisher Exact test. All statistical tests are two-sided. *Significance at a $p < 0.05$ level. [c]Ki67 global scoring method. ILC=invasive lobular carcinoma, IMC=invasive mucinous carcinoma, NST=invasive carcinoma of no special type, PR=progesterone receptor.

Table S7 Crosstabulation of Ki67 status and Prosigna risk group for Stratipath Breast low-risk cases (N=116).

		Ki67 status		
		**Low/Intermediate (<=29%)**	**High (>29%)**	**Total**
**Prosigna risk group**	**Low**	48 (54.55%)	4 (14.3%)	52 (44.83%)
	**Intermediate**	36 (40.90%)	16 (57.1%)	52 (44.83%)
	**High**	4 (4.55%)	8 (28.6%)	12 (10.34%)
	**Total**	88 (100%)	28 (100%)	116 (100%)

Table S8 Crosstabulation of Ki67 status and Prosigna risk group for Stratipath Breast high-risk cases (N=118).

		Ki67 status		
		**Low/Intermediate (<=29%)**	**High (>29%)**	**Total**
**Prosigna risk group**	**Low**	22 (32.4%)	2 (4.0%)	24 (20.3%)
	**Intermediate**	34 (50.0%	24 (48.0%)	58 (49.2%)
	**High**	12 (17.6%)	24 (48.0%)	36 (30.5%)
	**Total**	68 (100%)	50 (100%)	118 (100%)

Table S9 Comparison of agreement in risk stratification between Stratipath Breast risk group and Prosigna intrinsic subtype.

		Prosigna subtype		
		**Luminal A**	**Luminal B**	**Total**
**Stratipath risk group**	**Low**	83 (65.4%)	33 (30.8%)	116 (49.6%)
	**High**	44 (34.6%)	74 (69.2%)	118 (50.4%)
	**Total**	127 (100%)	107 (100%)	234 (100%)

Table S10 Comparison of agreement in risk stratification between Stratipath Breast risk group and Prosigna intrinsic subtype for grade 2 cases.

		Prosigna subtype		
		**Luminal A**	**Luminal B**	**Total**
**Stratipath risk group**	**Low**	71 (65.1%)	26 (38.8%)	97 (55.1%)
	**High**	38 (34.9%)	41 (61.2%)	79 (44.9%)
	**Total**	109 (100%)	67 (100%)	176 (100%)
